# Inflammation and Prediction of Death in Type 2 Diabetes. Evidence of an Intertwined Link With Tryptophan Metabolism

**DOI:** 10.1210/clinem/dgae593

**Published:** 2024-08-28

**Authors:** Claudia Menzaghi, Antonella Marucci, Mario Mastroianno, Giulio Di Ciaccia, Maria Pia Armillotta, Cornelia Prehn, Lucia Salvemini, Davide Mangiacotti, Jerzy Adamski, Andrea Fontana, Salvatore De Cosmo, Olga Lamacchia, Massimiliano Copetti, Vincenzo Trischitta

**Affiliations:** Research Unit of Diabetes and Endocrine Diseases, Fondazione Istituto di Ricovero e Cura a Carattere Scientifico “Casa Sollievo della Sofferenza,” 71013 San Giovanni Rotondo, Italy; Research Unit of Diabetes and Endocrine Diseases, Fondazione Istituto di Ricovero e Cura a Carattere Scientifico “Casa Sollievo della Sofferenza,” 71013 San Giovanni Rotondo, Italy; Scientific Direction, Fondazione Istituto di Ricovero e Cura a Carattere Scientifico “Casa Sollievo della Sofferenza,” 71013 San Giovanni Rotondo, Italy; Research Unit of Diabetes and Endocrine Diseases, Fondazione Istituto di Ricovero e Cura a Carattere Scientifico “Casa Sollievo della Sofferenza,” 71013 San Giovanni Rotondo, Italy; Research Unit of Diabetes and Endocrine Diseases, Fondazione Istituto di Ricovero e Cura a Carattere Scientifico “Casa Sollievo della Sofferenza,” 71013 San Giovanni Rotondo, Italy; Metabolomics and Proteomics Core, Helmholtz Zentrum München, German Research Center for Environmental Health, 85764 Neuherberg, Germany; Research Unit of Diabetes and Endocrine Diseases, Fondazione Istituto di Ricovero e Cura a Carattere Scientifico “Casa Sollievo della Sofferenza,” 71013 San Giovanni Rotondo, Italy; Research Unit of Diabetes and Endocrine Diseases, Fondazione Istituto di Ricovero e Cura a Carattere Scientifico “Casa Sollievo della Sofferenza,” 71013 San Giovanni Rotondo, Italy; Institute of Experimental Genetics, Helmholtz Zentrum München, German Research Center for Environmental Health, 85764 Neuherberg, Germany; Department of Biochemistry, Yong Loo Lin School of Medicine, National University of Singapore, Singapore 117597, Singapore; Institute of Biochemistry, Faculty of Medicine, University of Ljubljana, 1000 Ljubljana, Slovenia; Biostatistics Unit, Fondazione Istituto di Ricovero e Cura a Carattere Scientifico “Casa Sollievo della Sofferenza,” 71013 San Giovanni Rotondo, Italy; Unit of Internal Medicine, Fondazione Istituto di Ricovero e Cura a Carattere Scientifico “Casa Sollievo della Sofferenza,” 71013 San Giovanni Rotondo, Italy; Endocrinology Unit, Department of Medical and Surgical Sciences, University of Foggia, 71122 Foggia, Italy; Biostatistics Unit, Fondazione Istituto di Ricovero e Cura a Carattere Scientifico “Casa Sollievo della Sofferenza,” 71013 San Giovanni Rotondo, Italy; Research Unit of Diabetes and Endocrine Diseases, Fondazione Istituto di Ricovero e Cura a Carattere Scientifico “Casa Sollievo della Sofferenza,” 71013 San Giovanni Rotondo, Italy; Department of Experimental Medicine, Sapienza University of Rome, 00185 Rome, Italy

**Keywords:** inflammation risk score, IP-10, tryptophan pathway, prediction models, death risk

## Abstract

**Context:**

The role of inflammation in shaping death risk in diabetes is still unclear.

**Objective:**

To study whether inflammation is associated with and helps predict mortality risk in patients with type 2 diabetes. To explore the intertwined link between inflammation and tryptophan metabolism on death risk.

**Methods:**

There were 2 prospective cohorts: the aggregate Gargano Mortality Study (1731 individuals; 872 all-cause deaths) as the discovery sample, and the Foggia Mortality Study (490 individuals; 256 deaths) as validation sample. Twenty-seven inflammatory markers were measured. Causal mediation analysis and in vitro studies were carried out to explore the link between inflammatory markers and the kynurenine to tryptophan ratio (KTR) in shaping mortality risk.

**Results:**

Using multivariable stepwise Cox regression analysis, interleukin (IL)-4, IL-6, IL-8, IL-13, RANTES, and interferon gamma–induced protein-10 (IP-10) were independently associated with death. An inflammation score (I score) comprising these 6 molecules is strongly associated with death in both the discovery and the validation cohorts HR (95% CI) 2.13 (1.91-2.37) and 2.20 (1.79-2.72), respectively. The I score improved discrimination and reclassification measures (all *P* < .01) of 2 mortality prediction models based on clinical variables. The causal mediation analysis showed that 28% of the KTR effect on mortality was mediated by IP-10. Studies in cultured endothelial cells showed that 5-methoxy-tryptophan, an anti-inflammatory metabolite derived from tryptophan, reduces the expression of IP-10, thus providing a functional basis for the observed causal mediation.

**Conclusion:**

Adding the I score to clinical prediction models may help identify individuals who are at greater risk of death. Deeply addressing the intertwined relationship between low-grade inflammation and imbalanced tryptophan metabolism in shaping mortality risk may help discover new therapies targeting patients characterized by these abnormalities.

Type 2 diabetes is a leading cause of mortality (1, 2) and the source of a heavy personal and social load. As the prevalence of the disease is exploding (3), this burden will worsen and must therefore be faced with no hesitation.

The simultaneous and aggressive treatment of several risk factors reduces the rate of death in patients with type 2 diabetes (4). Attending diabetes clinics has also been reported to reduce the risk of mortality as a likely consequence of better and timely follow-up and treatment (5). Unfortunately, such an approach is expensive and laborious and therefore cannot be implemented in all individuals with diabetes. This makes it necessary to identify patients at high risk of death who would benefit most from optimal and multifaceted management.

The discovery of new disease markers is a way to improve the prediction of clinical events (6); furthermore, as an added value, the identification of novel markers can also reveal new pathogenic pathways that open the way to new therapies targeted at specific subgroups of patients (7).

Low-grade inflammation is known to shape the risk of total mortality in the general population (8) and in people with diabetes as well (9-12). Unfortunately, studies conducted on type 2 diabetes have investigated only a few markers (9-12), thus leaving the role of most inflammatory molecules unknown. Low-grade inflammation is a complex phenomenon that involves different paths that are often interconnected. We focused on some relevant paths, involving (1) adaptive immune cytokines related to T and or B lymphocytes; (2) chemokines that control cell migration on the inflammation site; and (3) growth factors, secreted after stimulation of cytokines and capable of regulating cell proliferation and differentiation. Accordingly, we investigated the role of several inflammatory molecules belonging to these 3 different paths (listed in Table S1, along with their principal sources and main functions (13)) on mortality in people with type 2 diabetes. Total mortality was used since the information on specific causes of death was not available. We then used the more robustly associated molecules with mortality to investigate whether a parsimonious index of their overall inflammatory activity improves prediction models of death in type 2 diabetes.

Given some previous evidence about the intertwined link between inflammation and tryptophan metabolism in several complex chronic diseases and survival probability (14-17), as a secondary aim, we also investigated this interdependence in relation to the risk of mortality in type 2 diabetes.

## Materials and Methods

### Participants

Two prospective cohorts of patients with type 2 diabetes (diagnosed according to American Diabetes Association 2003 criteria) (18) from Apulia, central-southern Italy, were investigated.

#### The aggregate Gargano Mortality Study—discovery sample

The aggregate Gargano Mortality Study (aGMS) consists of 2140 people with type 2 diabetes, recruited at the Endocrine Unit of IRCCS “Casa Sollievo della Sofferenza,” San Giovanni Rotondo, according to the same design and procedures from 2000 to 2008 (first period) and from 2008 to 2011 (second period) and prospectively followed until February 2023.

Serum inflammatory biomarkers were assessed in 1731 participants (80.9%), constituting the eligible sample for this analysis. Any analysis using data from aGMS was adjusted for the “recruitment period” (ie, first period = 0, second period = 1).

#### The Foggia Mortality Study—replication sample

The Foggia Mortality Study (FMS) comprises 1115 patients recruited consecutively at the Endocrine Unit of the University of Foggia from 2002 to 2008 and prospectively followed until March 2023. Serum inflammatory markers were assessed in 490 participants (43.9%), fully representative of the entire cohort, whose serum samples were available for this analysis.

For both cohorts, the only inclusion criterion was the presence of type 2 diabetes according to American Diabetes Association 2003 criteria (18) and the only exclusion criterion was the presence of poor life expectancy for nondiabetes-related diseases. The vital status of all participants was verified by interrogating the Italian Health Card Database upon data anonymization (https://sistemats1.sanita.finanze.it/wps/portal/).

Both prospective studies and the relative informed consent procedures were approved by the local Institutional Ethic Committees of IRCCS “Casa Sollievo della Sofferenza” and University of Foggia, respectively. All participants gave written informed consent.

### Measurement of Circulating Inflammatory Markers

All circulating biomarkers were collected at baseline and immediately stored at −80° until measurements. This procedure gave stable analyte concentrations (19, 20).

Circulating levels of 27 molecules were measured simultaneously in duplicate, using a multiplex detection 27-plex kit (Bio-Plex Pro Human Cytokine 27-plex assay, Bio-Rad Cat# M500KCAF0Y, RRID:AB_2893118) (Table S1 (13)). Three quality control samples (2 sex-mixed human serum samples from blood donors and 1 control serum provided by the manufacturer) were included in each plate. The median coefficient of variation (CV) was <15% for all analyzed markers, with the only exception of interferon-γ and vascular endothelial growth factor with CVs = 18.4% and 18.5%, respectively, and tumor necrosis factor-α = 24.2%. No values were out of the limit of detection. Data analyses were performed using Bio-Plex Manager software version 6.1 (Bio-Rad). Molecule concentrations were interpolated from an appropriate standard curve.

Tryptophan, kynurenine, and their ratio (KTR) were quantified using baseline fasting serum samples and the Absolute*IDQ* p180 Kit (BIOCRATES Life Sciences), as previously described (16, 17, 21). Sample handling was performed by a Hamilton Microlab STARTM robot (Hamilton Bonaduz AG, Bonaduz, Switzerland) and a Ultravap nitrogen evaporator (Porvair Sciences, Leatherhead, UK) and standard laboratory equipment. Mass spectrometric analyses were done on an API 4000 triple quadrupole system (SCIEX Deutschland GmbH, Darmstadt, Germany) equipped with a 1260 Series HPLC (Agilent Technologies Deutschland GmbH, Böblingen, Germany) and a HTC-xc PAL autosampler (CTC Analytics, Zwingen, Switzerland) controlled by the software Analyst 1.6.2. For the liquid chromatography, compounds were identified and quantified based on scheduled multiple reaction monitoring measurements, for the flow injection analysis part of multiple reaction monitoring measurements. Data evaluation for quantification of metabolite concentrations and quality assessment was performed with the software MultiQuant 3.0.1 (Sciex) and the MetIDQ software package. Metabolite concentrations were calculated using internal standards and reported in µM.

Serum high-sensitivity C-reactive protein (hs-CRP) was measured in duplicate, using a Multiplex Detection 4-Plex kit (Bio-Plex Pro Human Acute Phase 4-Plex Panel (Bio-Rad Cat# 171A4C09M, RRID:AB_3391717), also containing serum amyloid P component, α2-macroglobulin, and haptoglobin. The median CV was 15% for all proteins. Data analyses were performed using Bio-Plex Manager software version 6.1 (Bio-Rad). Serum hs-CRP concentrations were interpolated from an appropriate standard curve (11).

### Cell Culture

Human immortalized endothelial cells (TeloHAECs CRL-4052 from ATCC) were maintained at 37 °C and 5% CO_2_ in Vascular Cell Basal Medium (ATCC PCS-100-030), supplemented with Vascular Endothelial Cell Growth Kit (ATCC PCS-100-041). After amplification, cells were seeded in 6-well plates (Sigma-Aldrich) at ∼5 × 10^5^ cell per well. When 90% confluence was obtained, cell medium was changed with Vascular Cell Basal Medium plus 25 mM glucose for an additional 24 or 48 hours. In the last 12 hours of glucose incubation, 5-methoxy-DL-tryptophan (5-MTP) (Sigma-Aldrich) at a final concentration of 100 μM (or diluent) was added.

### RNA Extraction, cDNA Synthesis, and Gene Expression Analysis

Total RNA was isolated from cells by using RNeasy Mini kit (Qiagen). cDNA was generated by reverse transcription with iScript Reverse Transcription Supermix for RT-qPCR (BioRad) and used as template for subsequent analyses.

Expression levels of target *CXCL10* (C-X-C motif chemokine ligand 10), which encodes for the chemokine interferon gamma–induced protein 10 (IP-10) and housekeeping (*NONO*, *PPIA*, and *RPL13A*) genes were assessed by means of a predesigned SYBR green assay (BioRad assay ID: qHsaCED0034161, qHsaCED0002050, qHsaCED0038620, qHsaCED0020417) on an ABI-PRISM7900 (Applied Biosystems). The above 3 housekeeping genes were chosen from a list of a total of 30 analyzed genes because they were showing the best control gene stability (ie, M < 0.5) across samples and different experimental conditions (22) (data not shown).

*CXCL10* mRNA expression, normalized on the geometric mean of housekeeping genes, was calculated as the 2−ΔΔCT ratio on the 5.5 mM glucose plus 19.5 mM mannitol-treated cells at 24 hours, and used as the reference.

### Statistical Analysis

Patients’ baseline characteristics were reported as mean ± SD and frequency and percentage for continuous and categorical variables, respectively. Because of skewed distribution and for comparability between different biomarkers, their concentrations were logarithmically transformed and then standardized (ie, Z score for log). All covariates with missing values <5% were imputed by the random forest method (23).

Correlations between biomarkers were assessed using the Pearson correlation coefficient.

In both studies, the time variable was defined as the time between the baseline examination and date of the event (ie, all-cause mortality) or, for subjects who did not experience the event, the date of the last available clinical follow-up. Incidence rate for all-cause mortality was expressed as the number of events per 100 person-years.

To assess the association between serum inflammatory markers levels and all-cause mortality in the discovery sample (ie, aGMS), Bonferroni adjustment for multiple comparisons was used to determine the significance threshold in a Cox proportional hazards model adjusted for the recruitment period (0 or 1). The proportional hazards assumptions were tested through Schoenfeld residuals for each analysis.

Inflammatory markers that were significantly associated with mortality after Bonferroni adjustment, were further evaluated in a fully adjusted model comprising age at recruitment, gender, smoking habit, body mass index (BMI), diabetes duration, glycated hemoglobin (HbA1c), estimated glomerular filtration rate (eGFR), ongoing treatments, and recruitment period. Furthermore, molecules survived at this last step entered jointly a Cox model using a forward–backward stepwise analysis in order to minimize potential multicollinearity issues. Finally, a weighted inflammation score (I score) using the markers as selected by the stepwise analysis was created. The I score was obtained from the sum of biomarker values, each weighted by the regression coefficient from the forward–backward stepwise Cox model (eg, [0.364 × Z score for log_IP10] + [−0.246 × Z score for log_IL-13] + [0.244 × Z score for log_IL-6] + [0.264 × Z score for log_RANTES] + [−0.102 × Z score for log_IL-4] + [0.089 × Z score for log_IL-8]) divided by its SD and finally expressed per 1 SD increase. In addition, via recursive partitioning for survival trees (24), we investigated exploratory ranges of I scores which identify subgroups of patients at different risk of death.

As possible modifiers of the effect of the I score on mortality rate, age at recruitment, gender, smoking habits, BMI, diabetes duration, HbA1c, and eGFR were investigated. These variables were treated as continuous and also, to help clinical interpretation, as categorical. Cox proportional hazards analyses stratified according to the variables mentioned above along with the presence of multiplicative interaction terms were used.

To examine whether the validated I score in the 2 study samples pooled together increases the accuracy of both 6- and 10-year all-cause mortality prediction in type 2 diabetes, 2 different well-established models were utilized: ENFORCE (25, 26) and RECODe (27, 28), respectively. To pursue this aim, 346 and 592 events were available at 6 and 10 years of follow-up, respectively. Discrimination was measured by survival *c* statistics (29) while improvement in discrimination was assessed by delta *c* statistics and the survival version of the relative integrated discrimination improvement (rIDI) (30). In addition, the survival version of the category-free net reclassification improvement (cNRI), which examines whether the predicted probabilities of individuals with and without events move in the right direction (upward and downward, respectively) from the base to the new model, was evaluated (31). The 95% CIs for discrimination and reclassification measures were computed by bootstrap.

To test the possible intertwined link between inflammation and tryptophan metabolism, a causal mediation analysis was performed (32). Firstly, we checked if the kynurenine to tryptophan ratio (KTR) was associated with all-cause mortality (using a fully adjusted Weibull regression model) and with the 6 biomarkers firmly associated with all-cause mortality (using a fully adjusted linear regression model). Then, a causal mediation analysis was conducted using the “mediate” function defined in the “mediation” package in R. This function provided non-parametric estimates of the total, direct (ie, directly attributable to the exposure), and indirect effects, based on 1000 bootstrap replications, along with bias-corrected and accelerated 95% CIs. The percentage of mediated effect is defined as the ratio of the indirect to the total effect × 100, and its 2-sided empirical *P* value was defined as twice the number of times the percentages were negative out of the total number of bootstrap replications. Previous data make it possible to hypothesize a bidirectional link between inflammation and tryptophan metabolism in shaping the risk of cardiovascular disease (CVD) and survival probability. We then investigated 2 separate causal mediation models. One model included each cytokine as a mediator in the relationship between KTR and all-cause mortality, while the other included KTR as a mediator in the relationship between each cytokine and all-cause mortality.

*P* < .05 was considered to be statistically significant. All analyses were performed using SAS 9.4 (SAS Institute, Cary, NC) and R software (R Core Team, 2021).

## Results

Clinical features of patients from the aGMS and the FMS are summarized in Table 1. In the aGMS (left), 842 deaths occurred during a 13.1-year mean follow-up (22 676 person-years), while in the FMS (right) 256 deaths were observed during a mean follow-up of 12 years (5880 person-years).

**Table 1.** Clinical features of the 2 independent study cohorts

	aGMS, n = 1731	FMS, n = 490
Women (n) (%)	779 (45.0)	244 (49.8)
Age at recruitment (yrs)	62.5 ± 9.3	62.8 ± 11.6
Smoking habit (n) (%)	246 (14.2)	77 (15.7)
Diabetes duration (yrs)	11.6 ± 9.1	12.7 ± 9.7
BMI (kg/m^2^)	31.0 ± 5.7	30.1 ± 5.7
HbA_1c_ (%)	8.4 ± 1.9	9.0 ± 2.1
eGFR (mL/minute/1.73 m^2^)	80.2 ± 19.6	80.4 ± 25.5
Anti-hypertension therapy (n) (%)	1189 (68.7)	352 (71.8)
Insulin therapy (n) (%)	782 (45.2)	164 (33.5)
Statin therapy (n) (%)	890 (51.4)	150 (30.6)
Follow-up (yrs); (py)	13.1 ± 5.6 (22 676)	12.0 ± 5.5 (5880)
All-cause death (n) (%)	842 (48.6)	256 (52.2)
IR (n events per 100 py)*^a^* (95% CI)	3.2 (2.9-3.5)	3.7 (3.0-4.3)

Continuous variables are reported as mean ± SD and categorical variables as total frequencies and percentages.

Abbreviations: aGMS, aggregate Gargano Mortality Study; eGFR, estimated glomerular filtration rate (calculated using CKD-EPI equation); FMS, Foggia Mortality Study; HbA1c, glycated hemoglobin A1c; IR, incidence rate of all-cause death; py, person-years.

^*a*^Adjusted for age and sex.

### Association Between Inflammatory Markers and all-Cause Mortality

**Figure 1.**
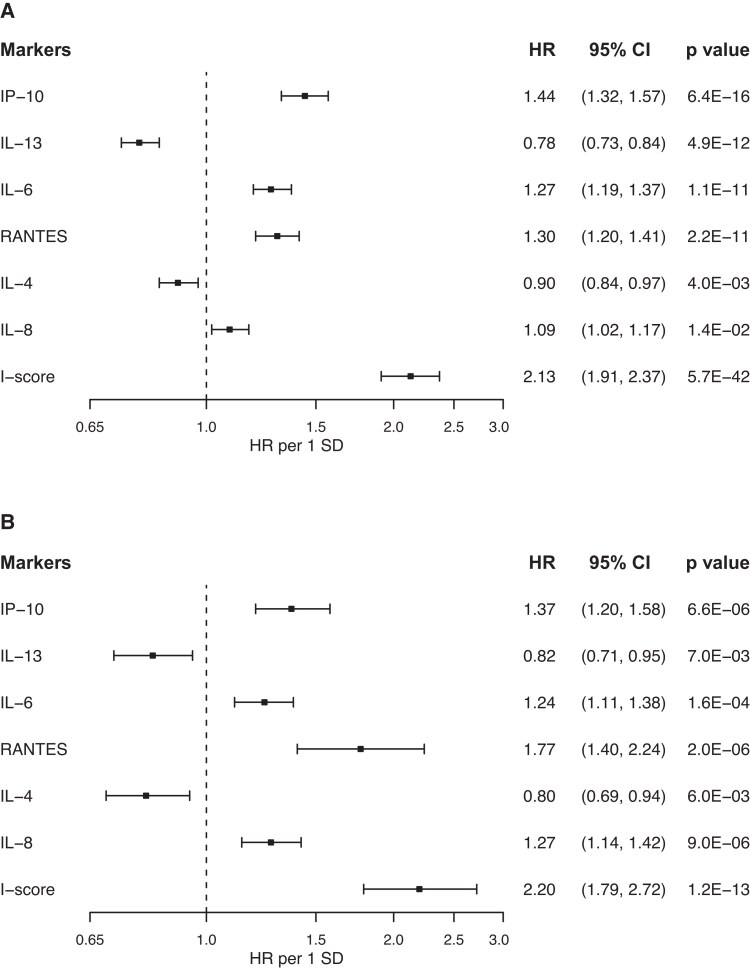
Associations between inflammatory markers, I score, and all-cause mortality. Hazard ratios (HRs) and 95% CIs per 1 SD increase of each inflammatory marker and the I score in aGMS (A) and FMS (B) were estimated in Cox regression models, adjusting for age at recruitment, gender, smoking habit, BMI, diabetes duration, HbA1c, eGFR, and ongoing treatments. For aGMS, the “recruitment period” was also included into the model.

In the aGMS, 17 of the 27 markers analyzed were significantly associated with all-cause mortality, having a *P* value below the threshold derived from the Bonferroni correction (.05/27 = 1.85 × 10^−3^) (Table 2, left). Eleven of these molecules, all with a CV <15%, remained independently associated with all-cause mortality in a fully adjusted model including age at recruitment, gender, smoking habit, BMI, diabetes duration, HbA1c, eGFR, all ongoing treatments, and recruitment period (8 with increased and 3 with decreased risk; Table 2, right). The 11 independently associated markers showed a pairwise correlation ranging from −0.20 to 0.92 (Fig. S1 (13)) with 6 of them surviving a further stepwise (forward–backward) procedure (Fig. 1A). The I score, created by summing the standardized serum values of these 6 molecules (see Materials and Methods) was strongly and independently associated with all-cause mortality in the same fully adjusted model as above (Fig. 1A and Table 3).

**Table 2.** Association between inflammatory markers and all-cause mortality in the aGMS

Unadjusted model*^a^*				Fully adjusted model*^b^*		
Cytokines	HR	95% CI	*P*	HR	95% CI	*P*
RANTES	1.67	1.53-1.82	6.2 × 10^–30^	**1.38**	**1.27-1.50**	**1.6** × 10**^–14^**
IL-6	1.33	1.26-1.41	1.5 × 10^–28^	**1**.**28**	**1.21-1.37**	**7.7** × 10**^–15^**
IP-10	1.68	1.53-1.84	4.4 × 10^–27^	**1**.**47**	**1.34-1.61**	**7.9** × 10**^–16^**
IL-13	0.70	0.65-0.75	1.6 × 10^–23^	**0**.**80**	**0.74-0.86**	**3.7** × 10**^–9^**
IL-8	1.32	1.24-1.40	5.5 × 10^–19^	**1**.**17**	**1.10-1.25**	**2.0 × 10^–6^**
FGF basic	1.31	1.23-1.40	4.4 × 10^–17^	**1**.**18**	**1.11-1.26**	**4.6 × 10^–7^**
IL-1ra	1.25	1.17-1.23	8.1 × 10^–12^	**1**.**17**	**1.10-1.25**	**9.6 × 10^–7^**
IL-15	0.81	0.76-0.87	3.9 × 10^–9^	**1**.**15**	**1.07-1.24**	**1.9 × 10^–4^**
MIP-1β	1.17	1.10-1.25	7.5 × 10^–7^	**1**.**13**	**1.06-1.20**	**2.1 × 10^–4^**
IL-4	0.87	0.82-0.92	2.0 × 10^–6^	**0**.**89**	**0.83-0.95**	**1.0 × 10^–3^**
PDGF-BB	1.20	1.10-1.31	2.8 × 10^–5^	**0**.**92**	**0.87-0.98**	**1.4 × 10^–2^**
IFN-γ	1.14	1.06-1.23	4.0 × 10^–4^	1.09	0.99-1.19	5.3 × 10^–1^
Eotaxin	1.17	1.08-1.25	4.1 × 10^–5^	1.06	0.99-1.14	8.3 × 10^–1^
IL-5	0.88	0.82-0.95	4.3 × 10^–4^	0.87	0.84-1.00	7.8 × 10^–1^
MIP-1α	1.12	1.04-1.19	1.0 × 10^–3^	1.05	0.98-1.12	1.5 × 10^–1^
IL-7	0.90	0.84-0.96	1.0 × 10^–3^	0.94	0.88-1.00	6.9 × 10^–1^
G-CSF	0.89	0.84-0.95	1.0 × 10^–3^	1.07	0.99-1.15	8.7 × 10^–1^
*IL-10*	*0*.*90*	*0.85-0.96*	*2.0 × 10^–3^*			
*IL-2*	*1*.*12*	*1.04-1.20*	*2.0 × 10^–3^*			
*IL-17A*	*1*.*08*	*1.04-1.16*	*2.1 × 10^–2^*			
*TNF-α*	*1*.*11*	*1.03-1.19*	*5.0 × 10^–3^*			
*IL-12 (p70)*	*1*.*07*	*0.99-1.17*	*5.0 × 10^–2^*			
*IL-9*	*1*.*04*	*0.94-1.15*	*6.9 × 10^–2^*			
*MCP-1 (MCAF)*	*1*.*06*	*0.99-1.14*	*8.7 × 10^–2^*			
*GM-CSF*	*1*.*06*	*098-1.14*	*1.2 × 10^–1^*			
*IL-1β*	*1*.*05*	*0.98-1.11*	*1.4 × 10^–1^*			
*VEGF*	*0*.*98*	*0.91-1.05*	*5.2 × 10^–1^*			

HR 95% CI are given for SD increase in inflammatory markers levels.

In italic are molecules that are not significantly associated with all-cause mortality in univariate analyses, after Bonferroni correction for multiple comparisons (*P* threshold = 1.85 × 10-3).

In bold are molecules significantly associated with all-cause mortality in the fully adjusted model.

Abbreviations: aGMS, aggregate Gargano Mortality Study; G-CSF, Granulocyte-Colony stimulating factor; GM-CSF, Granulocyte-macrophage colony-stimulating factor; FGF basic, Fibroblast Growth Factor; IFN-γ, interferon-gamma; IL, interleukin; IL-12 (p70), Interleukin-12p70; IL-1ra, Interleukin-1 receptor agonist; IP-10, interferon gamma–induced protein 10; MCAF, monocyte chemotactic and activating factor; MCP-1, monocyte chemoattractant protein-1; MIP, macrophage, inflammatory protein; PDGF-BB, platelet-derived growth factor-BB; RANTES, Regulated on Activation, Normal T cell Expressed and Secreted; TNF-α, tumor necrosis factor-alfa; VEGF, vascular endothelial growth factor.

^*a*^This analysis was adjusted only for the recruitment period, as described in Materials and Methods.

^*b*^This analysis was fully adjusted for age at recruitment, sex, smoking habit, BMI, HbA1c, eGFR, diabetes duration, ongoing treatments, and recruitment period.

**Table 3.** Multivariable associations with all-cause mortality in the aGMS (n = 1731)

	HR	95% CI		*P*
I score	2.13	1.91	2.37	5.7 × 10^−42^
Males vs females	1.39	1.19	1.61	1.9 × 10^−5^
Age at recruitment (per 10 years)	2.04	1.88	2.26	9.4 × 10^−44^
Smoking habit (yes vs no)	1.72	1.39	2.12	5.7 × 10^−7^
BMI (per 1 unit)	1.02	1.00	1.03	1.60 × 10^−2^
HbA1c (per 1 unit)	1.02	1.03	1.11	1.00 × 10^−2^
Disease duration (per 10 years)	1.02	0.94	1.13	5.6 × 10^−1^
eGFR (per 10 mL/min/m^2^)	1.12	1.07	1.17	5.2 × 10^−7^
Antihypertension therapy (yes vs no)	1.29	1.08	1.54	4.0 × 10^−3^
Insulin therapy (yes vs no)	1.48	1.25	1.74	4.0 × 10^−6^
Statins therapy (yes vs no)	0.92	0.79	1.06	2.5 × 10^−1^

HR (95% CI) for Inflammation score is given for 1 SD increase.

Analyses were adjusted for study cohort.

Abbreviations: aGMS, aggregate Gargano Mortality Study; BMI, body mass index; eGFR, estimated glomerular filtration rate (calculated using the CKD-EPI equation); HbA1c, glycated hemoglobin A1c; I score, inflammation score.

The relationship between all-cause mortality with each single marker as well as the I score (weighted according to the same effect sizes of each marker in the aGMS) was validated in the FMS, a totally independent sample using the same fully adjusted model as before (Fig. 1B). When hs-CRP levels were added into the model the association between the I score and death rate did not change (HR moving from 2.20, 95% CI 1.79-2.72 to 2.47, 1.95-3.17; *P* for HR heterogeneity = .47).

**Figure 2.**
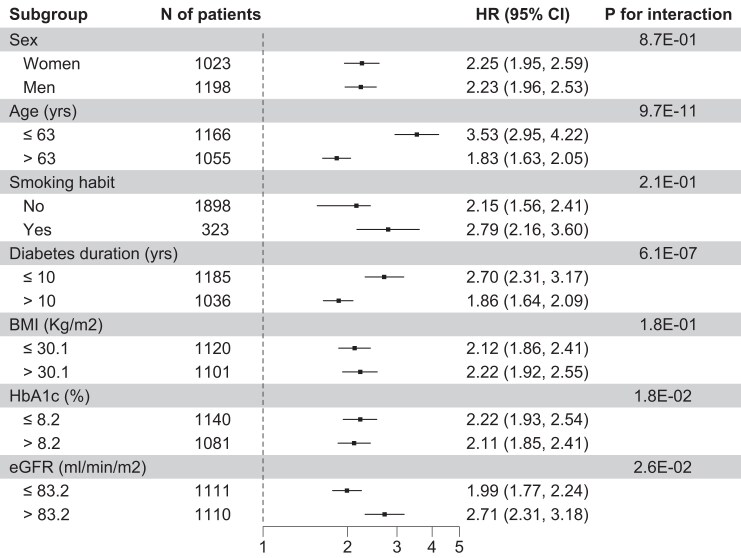
Associations between the I score and all-cause mortality in the combined sample (aGMS + FMS) by subgroups of demographical and clinical features. Hazard ratios (HRs) and 95% CI per 1 SD of I score increase were estimated by Cox regression models, adjusting for age at recruitment, gender, smoking habit, BMI, diabetes duration, HbA1c, eGFR, ongoing treatments, and study sample. The *P* value for interaction is shown for each subgroup.

In the 2 cohorts pooled together, patients still alive at the end of follow-up (N = 1123) have an I score = −0.31, while patients who were deceased (n = 1098) have an I score = 0.31 (*P* = 2.9 × 10^−106^). In this pooled sample, the association between the I score and all-cause mortality was significantly modified by age at recruitment, diabetes duration, HbA1c, and eGFR (*P* for interaction = 9.7 × 10^−11^, 6.1 × 10^−7^, 1.8 × 10^−2^, and 2.6 × 10^−2^, respectively). Subgroup analysis carried out to help clinical interpretability showed that the association between the I score and all-cause mortality was significantly and highly stronger in younger patients (ie, age below the median value of the entire cohort) (Fig. 2). Evidence of interaction in subgroup analysis was also observed with disease duration, HbA1c, and eGFR levels (ie, stronger effect in individuals with shorter duration, better glycemic control, and higher filtration rate, respectively) (Fig. 2).

In order to provide I score thresholds with clinical utility, patient subgroups at different risks for mortality have been identified through a survival tree analysis (Fig. S2 (13)). The subjects with I scores <−0.729 and ≥0.708 had the lowest (0.80 per 100 person-years, 95% CI 0.57-1.13) and the highest (11.02 per 100 person-years, 95% CI 9.74-12.47) incidence rate of mortality, respectively. Additional score thresholds that identify groups with intermediate risk of death are shown elsewhere (Fig. S2 (13)).

### Improvement of Established Prediction Models of Mortality

In the 2 pooled cohorts, the discrimination ability (*c* statistics) for all-cause mortality was 0.77 (95% CI 0.74-0.79, 6-year prediction) and 0.77 (95% CI 0.75-0.78, 10-year prediction) for the ENFORCE (25, 26) and the RECODe (27, 28) clinical models, respectively (Table 4). The addition of the I score significantly improved the *c* statistics (delta *c* statistics) in both models (Table 4). In addition, other measures of discrimination (IDI and rIDI) and reclassification (cNRI) were ameliorated in both models (Table 4). To avoid an overoptimistic conclusion, we investigated the improvement in *c* statistics provided by the I score in the FMS alone (ie, the validation cohort which was not used to discover the associated markers); similarly to what was observed in the pooled analysis, the delta *c* statistic was 0.03 (95% CI 0.01-0.05, *P* = 2 × 10^−4^, 6-year prediction) and 0.03 (95% CI 0.02-0.05, *P* = 7.5 × 10^−6^, 10-year prediction), at the top of ENFORCE and RECODe, respectively.

**Table 4.** Prediction of all-cause mortality by I score and by ENFORCE and RECODe (without and with the I score)

	Prediction models	Discrimination				Reclassification		
		*c* statistics (95% CI)	Δ*c* statistics (95% CI) *P* value	IDI *P* value	%rIDI *P* value	% ½ cNRI *P* value	% Events *P* value	% Nonevents *P* value
6-yr mortality	I score	0.71 (0.69-0.74)						
	ENFORCE	0.77 (0.74-0.79)						
	ENFORCE + I score	0.80 (0.78-0.82)	0.04 (0.02-0.05) 3 × 10^−8^	0.051 6 × 10^−10^	33.6 4 × 10^−9^	18.2 3 × 10^−11^	8.7 9 × 10^−2^	27.7 <2 × 10^−14^
10-yr mortality	I score	0.70 (0.68, 0.72)						
	RECODe	0.77 (0.75-0.78)						
	RECODe + I score	0.80 (0.78-0.81)	0.03 (0.02-0.04) 2 × 10^−13^	0.056 <2 × 10^−14^	27.2 <2 × 10^−14^	20.5 <2 × 10^−14^	11.5 4 × 10^−3^	29.5 <2 × 10^−14^

All *P values* are referred to comparisons vs the same base model (ie, with no I-score). Time horizon of 6 years for ENFORCE (n events = 346) and 10 years for RECODe (n events = 592).

Abbreviations: I score, inflammation score; cNRI, category-free net reclassification improvement; rIDI, relative integrated discrimination improvement.

Interestingly, very similar results were obtained on all prediction measures in both the ENFORCE and RECODe models with a parsimonious I score based only on the 4 most strongly associated inflammatory markers (ie, excluding IL-4 and IL-8) (Table S2 (13)).

In both prediction models, all improvements provided by the I score were particularly evident in younger patients (below the median age) (*P* < 0.0001 for all measures) compared with the improvements observed in older patients (see Table S3A and S3B (13)). This finding parallels the stronger association between the I score and the risk of death observed in younger subjects.

### The Link Between Inflammation and Tryptophan Metabolism in Shaping the Risk of Mortality

In a subset of 828 individuals from the aGMS which is fully representative of the entire cohort (clinical features shown in Table S4 (13)) the possible link between inflammation and tryptophan metabolism, by means of serum KTR, was investigated.

Of the 6 inflammatory markers identified and validated as associated with all-cause mortality, only IL-4, IL-6, IL-13, and IP-10 were also associated with the KTR (a prerequisite to run causal mediation analysis). These 4 molecules, as well as the KTR, were associated with all-cause mortality in this subset of individuals (Table S5, panel A (13)). The association with all-cause mortality when both KTR and each of the 4 markers were considered together is shown elsewhere (Panel B of Table S5 (13)). Most of the observed associations with mortality were virtually identical in the 2 alternative models, but that of KTR after adjustment for IP-10 (29% reduction in beta value) and those of IP-10 and IL-13 after adjustment for KTR (16% and 37% reduction in beta value, respectively).

The causal mediation analysis confirmed that more than one-quarter of the effect of KTR on mortality was mediated by IP-10 (28%, *P* = 8 × 10^−6^) and that conversely 16% (*P* = 6 × 10^−4^) of the effect of IP-10 is mediated by KTR. Furthermore, 39% of the effect of IL-13 on mortality was mediated by KTR (*P* = 2 × 10^−2^).

### Cell Culture Studies to get Insight on how IP-10 Mediates the Association Between KTR and Mortality

**Figure 3.**
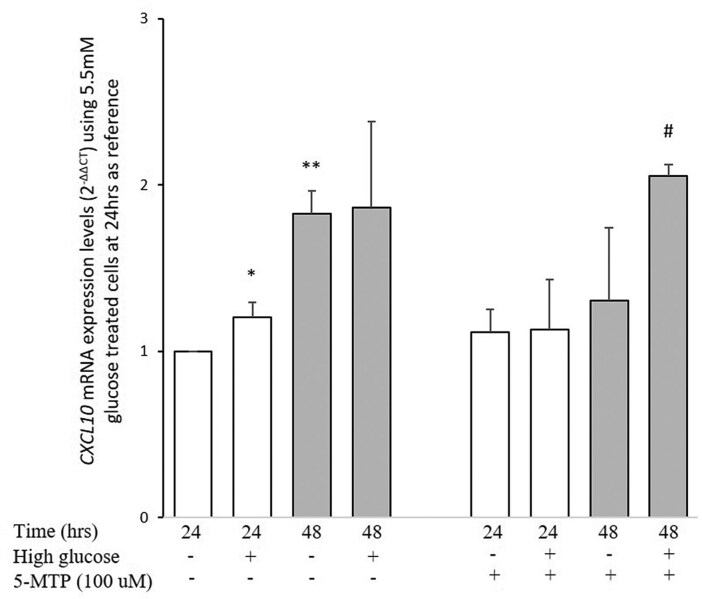
Glucose and 5-MTP induced C-X-C motif chemokine ligand 10 (*CXCL10)* mRNA expression. Bars represent *CXCL10* mRNA expression (which encodes for the chemokine IP-10), calculated as indicated in Materials and Methods, induced by high glucose (25 mM) concentration with or without 100 μM 5-MTP in cells incubated for 24 (white bars) and 48 (grey bars) hours. Data are means ± SD of 3 independent experiments. *Second vs first bar *P* = 1.8 × 10^−2^; **third vs first bar *P* = 4.5 × 10^−4^; #eighth vs seventh bar *P* = 4.2 × 10^−2^.

We tested in TeloHAECs whether 5-MTP, a 5-OH-tryptophan–derived metabolite with anti-inflammatory activity (33, 34), influences the expression of IP-10 under different experimental conditions combining short- and long-term incubation at low- and high-glucose concentrations. Indeed, compared with that in cells after a 24-hour incubation at 5 mM glucose, taken as the control condition, the expression of *CXCL10*, which encodes for IP-10, was slightly but significantly increased by a 25 mM glucose concentration at 24 hours (*P* = 1.8 × 10^−2^; Fig. 3, left half, white bars) and almost doubled by a 48-hour incubation (*P* = 4.5 × 10^−4^; Fig. 3 right half, grey bars). At this time point, no further stimulatory effect of a 25 mM glucose concentration was observed. Coincubation with 100 µM 5-MTP abrogated the stimulation of IP-10 expression exerted by a high glucose concentration at 24 hours (Fig. 3, left) as well as by prolonged incubation at 48 hours, thus restoring the stimulatory effect of high glucose concentration (*P* = 4E^−2^) (Fig. 3, right).

## Discussion

We investigated comprehensively the role of low-grade inflammation on all-cause mortality in 2221 individuals with type 2 diabetes, from 2 different cohorts. Indeed, 27 inflammatory markers were measured, with 6 of them (IL-4, IL-6, IL-8, IL-13, RANTES, and IP-10) independently associated in the first discovery sample and then confirmed in the second validation sample. Of note, the I score, comprising these 6 markers, was associated with all-cause death in both the discovery and the validation sample with an effect similar in magnitude to that of being 10 years older and much stronger than that of gender, kidney function, essential hypertension, and the severity of diabetes, as indicated by insulin treatment. Notably, the association with mortality rate of the I score was independent of hs-CRP (11), suggesting that the 2 inflammatory markers provide information on different pathways related to premature death in type 2 diabetes.

The association between I score and all-cause mortality was much stronger in younger individuals and in those with shorter duration of diabetes, meaning that the deleterious effect of chronic inflammation on the probability of survival is more evident when other risk factors such as age and long disease duration are less prevalent. This suggests the need to prevent or treat inflammation in the early stages of type 2 diabetes, especially in relatively young people in whom diabetes has the greatest negative effect on life expectancy (1) and in whom intervention strategies have more time to improve the trajectory of the disease's fearsome consequences.

Importantly from a clinical point of view, the I score improved discrimination measures of ENFORCE and RECODe, 2 well-established prediction models of all-cause death in type 2 diabetes which are based on clinical variables (25-28). Although statistically significant, the improvement of the *c* statistics is rather small, but it is worth noticing that in already well-performing models, as are those we used here, this index lacks sensitivity in detecting further discrimination improvements (35). In both models, the percentage rIDI, an alternative discrimination index, is higher than the threshold requested by international guidelines for adding new biomarkers on top of established prediction models (36). In addition, adding the I score to both models correctly reclassified a significant proportion of individuals, especially nonevents, thus reducing the risk of overestimation. Finally, in both models, the improvement of discrimination and reclassification measures provided by the I score was particularly evident in patients with relatively younger age. Overall, our findings advocate the use of the I score on top of ENFORCE or RECODe to improve the identification of individuals with type 2 diabetes at highest risk of death who would benefit most from aggressive, laborious, and costly management that cannot (and probably does not need to) be implemented in all individuals with diabetes.

The I score includes both proinflammatory (IL-6 and IL-8) and anti-inflammatory (IL-4 and IL-13) inflammatory cytokines as well as 2 chemokines (RANTES or CCL5 and IP-10 or CXCL10). IL-6 has previously been studied as a predictor of CVD (37) and mortality (38) also in patients with type 2 diabetes (10, 39). IL-6 mediates humoral and cellular responses (40) and is closely related to IL-8, a neutrophil chemotactic proinflammatory cytokine also associated with (CVD) and involved in mitogenesis, angiogenesis inhibition, and leukocyte activation (41).

The anti-inflammatory IL-4 and IL-13 cytokines share many biological and immunoregulatory functions on B lymphocytes, monocytes, dendritic cells, and fibroblasts (42). Generally, IL-4/-13 signaling to peripheral tissues promotes cell proliferation, which mediates beneficial responses (43). IL-4 circulating levels are decreased in elderly people (44) and low levels of IL-13 are associated with increased mortality risk in diabetes (17).

Finally, chemokines RANTES and IP-10, which act on endothelial and vascular smooth muscle cells, are postulated to be involved in the development and manifestation of atherosclerosis and CVD (45, 46).

In type 2 diabetes, tryptophan metabolism is associated with all-cause mortality (17). In detail, serum tryptophan entails a high survival rate while, in contrast, serum kynurenine and the KTR are positively related to the risk of death (17).

Indeed, KTR is a marker of tryptophan downstream metabolism that recognizes 2 alternative pathways: one that through kynurenine is proinflammatory (47) and another that synthesizes 5-MTP, an anti-inflammatory metabolite derived from 5-OH-tryptophan (33, 34). Causal mediation analysis showed that IP-10 mediates part of the deleterious effect of KTR on the risk of death. A possible mechanism underlying this effect is, at least partially, indicated by our studies on cultured endothelial cells showing that under several experimental conditions IP-10 expression is controlled by 5-MTP, thus making this chemokine central for the anti-inflammatory effect of tryptophan metabolism. Conversely, KTR mediates part of the effect on all-cause mortality of IP-10, suggesting a binary mediation between the 2 risk factors. KTR also mediates part of the effect of IL-13, confirming our previous observation obtained in a smaller sample (17). As a whole, our data indicate the existence of a pathway that through an intertwined relationship between low-grade inflammation and tryptophan metabolism shapes the risk of death in people with diabetes, thus opening new research routes to discover tailor-made treatments for “inflamed” patients with imbalanced tryptophan metabolism.

Our study has several strengths. We used a rigorous study design with discovery and replication cohorts prospectively analyzed with a clinical phenotype thoroughly investigated. The adopted pipeline to skim the most associated serum inflammatory markers with all-cause mortality included Bonferroni adjustment, confounders adjusted model, and stepwise analysis, a conservative approach that lowers the false-positive rate.

We also carried out a comprehensive analysis on a large number of quality-controlled inflammatory biomarkers, whereas previous studies on the role of inflammation on mortality in patients with type 2 diabetes have evaluated only a few markers (9-12). In addition, our present study tested the ability of an I score, based on the most robustly associated markers, to improve 2 well-established and validated prediction models (25-28), thus making our findings implementable in the real-life clinical setting. Finally, we gained information on the intertwined relationship between the inflammation and tryptophan pathways (14) whose link is a promising field of study aimed at paving the way for new therapies to treat individuals with type 2 diabetes (and perhaps also other chronic conditions) characterized by low-grade inflammation and imbalanced tryptophan metabolism.

We have also to recognize several limitations. Firstly, the 2 cohorts we studied are geographically close, thus limiting the generalizability of our finding. In addition, we lack data on cardiovascular mortality, thus making it impossible to look for the association between this specific cause of death and inflammation previously reported to be associated with some inflammatory markers in the general population (38). This said, it should also be considered that in individuals with diabetes cardiovascular mortality, although still important (48, 49), is no longer the leading cause of death, now representing only one-quarter of all events (50).

In conclusion, we have comprehensively investigated and characterized the association between inflammation and all-cause mortality in people with type 2 diabetes, which may have clinical implication in identifying individuals who are at greater risk of death. We also provided evidence that an intertwined relationship between inflammation and the tryptophan metabolism influences the rate of mortality. This may pave the way for new therapies targeted at subgroups of patients characterized by low-grade inflammation and imbalanced tryptophan metabolism.

## Data Availability

The data sets generated during and/or analyzed during the current study are available from the corresponding author upon reasonable request.

## Abbreviations

5-MTP: 5-methoxy-DL-tryptophan
aGMS: aggregate Gargano Mortality Study
BMI: body mass index
cNRI: category-free net reclassification improvement
CV: coefficient of variation
CVD: cardiovascular disease
eGFR: estimated glomerular filtration rate
FMS: Foggia Mortality Study
HbA1c: glycated hemoglobin
hs-CRP: high-sensitivity C-reactive protein
IL: interleukin
IP-10: interferon gamma–induced protein 10
I score: inflammation score
KTR: kynurenine to tryptophan ratio
RANTES: Regulated on Activation, Normal T cell Expressed and Secreted
rIDI: relative integrated discrimination improvement

## References

[1] Emerging Risk Factors Collaboration . Life expectancy associated with different ages at diagnosis of type 2 diabetes in high-income countries: 23 million person-years of observation. Lancet Diabetes Endocrinol. 2023;11(10):731‐742. 37708900 10.1016/S2213-8587(23)00223-1 PMC7615299

[2] Tomic D , Morton JI, Chen L, et al Lifetime risk, life expectancy, and years of life lost to type 2 diabetes in 23 high-income jurisdictions: a multinational, population-based study. Lancet Diabetes Endocrinol. 2022;10(11):795‐803. 36183736 10.1016/S2213-8587(22)00252-2 PMC10988609

[3] GBD 2021 Diabetes Collaborators . Global, regional, and national burden of diabetes from 1990 to 2021, with projections of prevalence to 2050: a systematic analysis for the global burden of disease study 2021. Lancet. 2023;402(10397):203‐234. 37356446 10.1016/S0140-6736(23)01301-6 PMC10364581

[4] Gæde P , Oellgaard J, Carstensen B, et al Years of life gained by multifactorial intervention in patients with type 2 diabetes mellitus and microalbuminuria: 21 years follow-up on the Steno-2 randomised trial. Diabetologia. 2016;59(11):2298‐2307. 27531506 10.1007/s00125-016-4065-6 PMC5506099

[5] Bonora E , Monami M, Bruno G, Zoppini G, Mannucci E. Attending diabetes clinics is associated with a lower all-cause mortality. a meta-analysis of observational studies performed in Italy. Nutr Metab Cardiovasc Dis. 2018;28(5):431‐435. 29627120 10.1016/j.numecd.2018.02.009

[6] Cook NR . Quantifying the added value of new biomarkers: how and how not. Diagn Progn Res. 2018;2(1):14. 31093563 10.1186/s41512-018-0037-2 PMC6460632

[7] Qiu S , Cai Y, Yao H, et al Small molecule metabolites: discovery of biomarkers and therapeutic targets. Signal Transduct Target Ther. 2023;8(1):132. 36941259 10.1038/s41392-023-01399-3 PMC10026263

[8] Furman D , Campisi J, Verdin E, et al Chronic inflammation in the etiology of disease across the life span. Nat Med. 2019;25(12):1822‐1832. 31806905 10.1038/s41591-019-0675-0 PMC7147972

[9] Landman GW , Kleefstra N, Groenier KH, et al Inflammation biomarkers and mortality prediction in patients with type 2 diabetes (ZODIAC-27). Atherosclerosis. 2016;250:46‐51. 27179179 10.1016/j.atherosclerosis.2016.04.015

[10] Lowe G , Woodward M, Hillis G, et al Circulating inflammatory markers and the risk of vascular complications and mortality in people with type 2 diabetes and cardiovascular disease or risk factors: the ADVANCE study. Diabetes. 2014;63(3):1115‐1123. 24222348 10.2337/db12-1625

[11] Scarale MG , Copetti M, Garofolo M, et al The synergic association of hs-CRP and serum amyloid P component in predicting all-cause mortality in patients with type 2 diabetes. Diabetes Care. 2020;43(5):1025‐1032. 32144164 10.2337/dc19-2489

[12] Scarale MG , Antonucci A, Cardellini M, et al A serum resistin and multicytokine inflammatory pathway is linked with and helps predict all-cause death in diabetes. J Clin Endocrinol Metab. 2021;106(11):e4350‐e4359. 34192323 10.1210/clinem/dgab472

[13] Menzaghi C , Marucci A, Mastroianno M, et al Inflammation and prediction of death in type 2 diabetes. Evidence of an intertwined link with tryptophan metabolism. Figshare. 10.6084/m9.figshare.26404324figshare. Date of deposit 22 August 2024. PMC12012783 39193712

[14] Tsuji A , Ikeda Y, Yoshikawa S, et al The tryptophan and kynurenine pathway involved in the development of immune-related diseases. Int J Mol Sci. 2023;24(6):5742. 36982811 10.3390/ijms24065742 PMC10051340

[15] Liu JJ , Ching J, Wee HN, et al Plasma tryptophan-kynurenine pathway metabolites and risk for progression to end-stage kidney disease in patients with type 2 diabetes. Diabetes Care. 2023;46(12):2223‐2231. 37796480 10.2337/dc23-1147 PMC10698226

[16] Trischitta V , Mastroianno M, Scarale MG, et al Circulating metabolites improve the prediction of renal impairment in patients with type 2 diabetes. BMJ Open Diabetes Res Care. 2023;11(5):e003422. 10.1136/bmjdrc-2023-003422 PMC10514631 37734903

[17] Scarale MG , Mastroianno M, Prehn C, et al Circulating metabolites associate with and improve the prediction of all-cause mortality in type 2 diabetes. Diabetes. 2022;71(6):1363‐1370. 35358315 10.2337/db22-0095

[18] American Diabetes Association . Standards of medical care in diabetes. Diabetes Care. 2004;27(Suppl 1):S15‐S35. 14693923 10.2337/diacare.27.2007.s15

[19] Stervbo U , Bajda S, Wehler P, et al Stability of 12 T-helper cell-associated cytokines in human serum under different pre-analytical conditions. Cytokine. 2020;129:155044. 32109722 10.1016/j.cyto.2020.155044

[20] Haid M , Muschet C, Wahl S, et al Long-term stability of human plasma metabolites during storage at -80 °C. J Proteome Res. 2018;17(1):203‐211. 29064256 10.1021/acs.jproteome.7b00518

[21] Zukunft S , Prehn C, Röhring C, et al High-throughput extraction and quantification method for targeted metabolomics in murine tissues. Metabolomics. 2018;14(1):18. 29354024 10.1007/s11306-017-1312-x PMC5748028

[22] Vandesompele J , De Preter K, Pattyn F, et al Accurate normalization of real-time quantitative RT-PCR data by geometric averaging of multiple internal control genes. Genome Biol. 2002;3(7):RESEARCH0034. 12184808 10.1186/gb-2002-3-7-research0034 PMC126239

[23] Stekhoven DJ , Bühlmann P. MissForest–non-parametric missing value imputation for mixed-type data. Bioinformatics. 2012;28(1):112‐118. 22039212 10.1093/bioinformatics/btr597

[24] Leo B , Jerome F, Olshen RA, Stone CJ. Classification and Regression Trees. Taylor&Francis Group; 1984:1‐368

[25] De Cosmo S , Copetti M, Lamacchia O, et al Development and validation of a predicting model of all-cause mortality in patients with type 2 diabetes. Diabetes Care. 2013;36(9):2830‐2835. 23637348 10.2337/dc12-1906 PMC3747924

[26] Copetti M , Shah H, Fontana A, et al Estimation of mortality risk in type 2 diabetic patients (ENFORCE): an inexpensive and parsimonious prediction model. J Clin Endocrinol Metab. 2019;104(10):4900‐4908. 31087060 10.1210/jc.2019-00215 PMC6734484

[27] Basu S , Sussman JB, Berkowitz SA, Hayward RA, Yudkin JS. Development and validation of risk equations for complications of type 2 diabetes (RECODe) using individual participant data from randomised trials. Lancet Diabetes Endocrinol. 2017;5(10):788‐798. 28803840 10.1016/S2213-8587(17)30221-8 PMC5769867

[28] Basu S , Sussman JB, Berkowitz SA, et al Validation of risk equations for complications of type 2 diabetes (RECODe) using individual participant data from diverse longitudinal cohorts in the U.S. Diabetes Care. 2018;41(3):586‐595. 29269511 10.2337/dc17-2002 PMC5829967

[29] Uno H , Tian L, Cai T, Kohane IS, Wei LJ. A unified inference procedure for a class of measures to assess improvement in risk prediction systems with survival data. Stat Med. 2013;32(14):2430‐2442. 23037800 10.1002/sim.5647 PMC3734387

[30] Pencina MJ , D'Agostino RB Sr, D'Agostino RB Jr, Vasan RS. Evaluating the added predictive ability of a new marker: from area under the ROC curve to reclassification and beyond. Stat Med. 2008;27(2):157‐172. discussion 207-112. 17569110 10.1002/sim.2929

[31] Pencina MJ , D'Agostino RB Sr, Steyerberg EW. Extensions of net reclassification improvement calculations to measure usefulness of new biomarkers. Stat Med. 2011;30(1):11‐21. 21204120 10.1002/sim.4085 PMC3341973

[32] Imai K , Keele L, Tingley D. A general approach to causal mediation analysis. Psychol Methods. 2010;15(4):309‐334. 20954780 10.1037/a0020761

[33] Chen DQ , Cao G, Chen H, et al Identification of serum metabolites associating with chronic kidney disease progression and anti-fibrotic effect of 5-methoxytryptophan. Nat Commun. 2019;10(1):1476. 30931940 10.1038/s41467-019-09329-0 PMC6443780

[34] Wang YF , Hsu YJ, Wu HF, et al Endothelium-derived 5-methoxytryptophan is a circulating anti-inflammatory molecule that blocks systemic inflammation. Circ Res. 2016;119(2):222‐236. 27151398 10.1161/CIRCRESAHA.116.308559

[35] Cook NR . Use and misuse of the receiver operating characteristic curve in risk prediction. Circulation. 2007;115(7):928‐935. 17309939 10.1161/CIRCULATIONAHA.106.672402

[36] Goff DC Jr , Lloyd-Jones DM, Bennett G, et al 2013 ACC/AHA guideline on the assessment of cardiovascular risk: a report of the American College of Cardiology/American Heart Association task force on practice guidelines. J Am Coll Cardiol. 2014;63(25 Pt B):2935‐2959. 24239921 10.1016/j.jacc.2013.11.005 PMC4700825

[37] Fisman EZ , Tenenbaum A. The ubiquitous interleukin-6: a time for reappraisal. Cardiovasc Diabetol. 2010;9(1):62. 20937099 10.1186/1475-2840-9-62 PMC2959009

[38] Sattar N , Murray HM, Welsh P, et al Are markers of inflammation more strongly associated with risk for fatal than for nonfatal vascular events? PLoS Med. 2009;6(6):e1000099. 19554082 10.1371/journal.pmed.1000099 PMC2694359

[39] Ofstad AP , Gullestad L, Orvik E, et al Interleukin-6 and activin A are independently associated with cardiovascular events and mortality in type 2 diabetes: the prospective asker and bærum cardiovascular diabetes (ABCD) cohort study. Cardiovasc Diabetol. 2013;12(1):126. 23987834 10.1186/1475-2840-12-126 PMC3766106

[40] Tanaka T , Narazaki M, Kishimoto T. IL-6 in inflammation, immunity, and disease. Cold Spring Harb Perspect Biol. 2014;6(10):a016295. 25190079 10.1101/cshperspect.a016295 PMC4176007

[41] Apostolakis S , Vogiatzi K, Amanatidou V, Spandidos DA. Interleukin 8 and cardiovascular disease. Cardiovasc Res. 2009;84(3):353‐360. 19617600 10.1093/cvr/cvp241

[42] Bhattacharjee A , Shukla M, Yakubenko VP, Mulya A, Kundu S, Cathcart MK. IL-4 and IL-13 employ discrete signaling pathways for target gene expression in alternatively activated monocytes/macrophages. Free Radic Biol Med. 2013;54:1‐16. 23124025 10.1016/j.freeradbiomed.2012.10.553 PMC3534796

[43] Bakhshian Nik A , Alvarez-Argote S, O'Meara CC. Interleukin 4/13 signaling in cardiac regeneration and repair. Am J Physiol Heart Circ Physiol. 2022;323(5):H833‐H844. 36149768 10.1152/ajpheart.00310.2022 PMC9602781

[44] Palmeri M , Misiano G, Malaguarnera M, et al Cytokine serum profile in a group of Sicilian nonagenarians. J Immunoassay Immunochem. 2012;33(1):82‐90. 22181823 10.1080/15321819.2011.601781

[45] Bakogiannis C , Sachse M, Stamatelopoulos K, Stellos K. Platelet-derived chemokines in inflammation and atherosclerosis. Cytokine. 2019;122:154157. 29198385 10.1016/j.cyto.2017.09.013

[46] van den Borne P , Quax PH, Hoefer IE, Pasterkamp G. The multifaceted functions of CXCL10 in cardiovascular disease. Biomed Res Int. 2014;2014:893106. 24868552 10.1155/2014/893106 PMC4017714

[47] Badawy AA . Kynurenine pathway of tryptophan metabolism: regulatory and functional aspects. Int J Tryptophan Res. 2017;10:1178646917691938. 28469468 10.1177/1178646917691938 PMC5398323

[48] Yun JS , Ko SH. Current trends in epidemiology of cardiovascular disease and cardiovascular risk management in type 2 diabetes. Metabolism. 2021;123:154838. 34333002 10.1016/j.metabol.2021.154838

[49] Ma CX , Ma XN, Guan CH, Li YD, Mauricio D, Fu SB. Cardiovascular disease in type 2 diabetes mellitus: progress toward personalized management. Cardiovasc Diabetol. 2022;21(1):74. 35568946 10.1186/s12933-022-01516-6 PMC9107726

[50] Pearson-Stuttard J , Buckley J, Cicek M, Gregg EW. The changing nature of mortality and morbidity in patients with diabetes. Endocrinol Metab Clin North Am. 2021;50(3):357‐368. 34399950 10.1016/j.ecl.2021.05.001

