# Correction to: “Inflammation and Prediction of Death in Type 2 Diabetes. Evidence of an Intertwined Link With Tryptophan Metabolism”

**DOI:** 10.1210/clinem/dgaf119

**Published:** 2025-03-07

In the above-named article by Menzaghi C, Marucci A, Mastroianno M, Di Ciaccia G, Armillotta MP, Prehn C, Salvemini L, Mangiacotti D, Adamski J, Fontana A, De Cosmo S, Lamacchia O, Copetti M, and Trischitta V (*J Clin Endo Metab*. 2024; doi: 10.1210/clinem/dgae593), there was an error in the Funding section.

In the originally published article, the first sentence in the Funding section read “This study was supported by Ministero dell’Istruzione dell’Università (PRIN) 2015 and by Ministero dell’Università PNRR M4C2I1.3 Heal Italia project PE00000019 grants (to V.T.) and by Ministero della Salute grant RF-2013-02356459 (to C.M.); PNRR-MAD-2022-12375970 (to C.M. and S.D.C.); Ricerca corrente 2022-2024 (C.M., A.M., S.D.C., V.T.) and European Health Data & Evidence Network (EHDEN) project (to C.M.).” The following information for the grant PNRR-MAD-2022-12375970 has been added: “funded by the European Union - Next Generation EU - NRRP M6C2 - Investment 2.1 Enhancement and strengthening of biomedical research in the NHS.” In the corrected article, the sentence now reads “This study was supported by Ministero dell’Istruzione dell’Università (PRIN) 2015 and by Ministero dell’Università PNRR M4C2I1.3 Heal Italia project PE00000019 grants (to V.T.) and by Ministero della Salute grant RF-2013-02356459 (to C.M.); PNRR-MAD-2022-12375970 funded by the European Union - Next Generation EU - NRRP M6C2 - Investment 2.1 Enhancement and strengthening of biomedical research in the NHS (to C.M. and S.D.C.); Ricerca corrente 2022-2024 (C.M., A.M., S.D.C., V.T.) and European Health Data & Evidence Network (EHDEN) project (to C.M.).”

The article has been corrected online.

doi: 10.1210/clinem/dgae593

